# Vaccine effectiveness in symptom and viral load mitigation in COVID-19 breakthrough infections in South Korea

**DOI:** 10.1371/journal.pone.0290154

**Published:** 2023-08-16

**Authors:** Jieun Jang, Hyopin Jeong, Bong-Hwa Kim, Sura An, Hye-Ryun Yang, Sunjoo Kim

**Affiliations:** 1 Gyeongnam Center for Infectious Disease Control and Prevention, Changwon-si, Gyeongnam, Republic of Korea; 2 Department of Laboratory Medicine, Gyeongsang National University College of Medicine, Health Science Institute, Jinju-si, Gyeongnam, Republic of Korea; 3 Department of Laboratory Medicine, Gyeongsang National University Changwon Hospital, Changwon-si, Gyeongnam, Republic of Korea; Lerner Research Institute - Cleveland Clinic, UNITED STATES

## Abstract

**Objectives:**

Severe acute respiratory syndrome coronavirus 2 (SARS-CoV-2) vaccine effectiveness in coronavirus disease (COVID-19) patients with breakthrough infections has not been established in South Korea. To address this, we assessed the impact of vaccination on symptom occurrence and viral load.

**Methods:**

We performed a retrospective cohort study of 9,030 COVID-19 patients enrolled between February and November 2021. The impact of vaccination on the incidence of symptoms and viral load as indicated by cycle threshold (Ct) values of *RdRp* and *E* genes was evaluated using relative risks (RRs) and 95% confidence intervals (95% CIs).

**Results:**

Compared with unvaccinated patients, fully vaccinated patients were associated with a reduced symptom onset of cough, sputum, and myalgia in COVID-19 patients (RR (95% CI) = 0.86 (0.75–0.99) for cough; RR (95% CI) = 0.74 (0.56–0.98) for sputum; RR (95% CI) = 0.65 (0.53–0.79) for myalgia, respectively). Additionally, lower risk of high viral load, Ct value of *RdRp* gene <15 or Ct value of *E* gene <15, was observed especially in fully vaccinated patients younger than 40 years ((RR (95% CI) = 0.69 (0.49–0.96) for *RdRp* gene; (RR (95% CI) = 0.71 (0.53–0.95) for *E* gene).

**Conclusion:**

SARS-CoV-2 vaccination was associated with a reduced risk of COVID-19 symptoms as well as decreased viral load, especially in patients younger than 40 years.

## Introduction

The first case of the novel coronavirus disease (COVID-19), caused by the severe acute respiratory syndrome coronavirus 2 (SARS-CoV-2), was reported in December 2019 in Wuhan city in the People’s Republic of China [1], and the COVID-19 pandemic was declared by the World Health Organization on 11 March 2020 [2]. More than 610,000,000 COVID-19 cases have been confirmed worldwide until 25 September 2022. COVID-19 has caused mortality, with more than 6.5 million deaths worldwide (1.1% of all confirmed cases) [3].

As a prophylactic response to the COVID-19 pandemic, several COVID-19 vaccines have been developed and have received emergency use authorization from the Unites States Food and Drug Administration (FDA) and the European Medicines Agency (EMA) [4–9].

Worldwide, vaccination against COVID-19 commenced and has been implemented at an unprecedented pace including in South Korea, which started COVID-19 vaccination in February 2021. In South Korea, four COVID-19 vaccines (BNT162b2, AZD1222, mRNA-1273, and Ad26.COV2.S) have been administered, and full-vaccination coverage was noted in approximately 80.0% of the population until November 2021 [10].

Vaccine efficacy of more than 80% was reported [11]; however, waning immunity to SARS-CoV-2 after vaccination [12–14] and the appearance of virus variants have both led to a decrease in vaccine effectiveness [15, 16].

In the situation where the vaccine effectiveness for prevention of infection is decreasing and the breakthrough infection are increasing, it is necessary to evaluate whether the additional effectiveness of the vaccine can be expected in addition to the prevention of infection. In other words, it is needed to evaluate whether vaccine is worthwhile even in COVID-19 patients with breakthrough infection, and mitigation of symptoms and decrease in viral load can be useful measure of vaccine effectiveness.

Therefore, this study was conducted to evaluate the impact of COVID-19 vaccines in preventing symptoms and decreasing viral loads with consideration of potential confounders using the real-world data including the overall confirmed COVID-19 cases in a province in Korea.

## Materials and methods

### Participants

This study was performed based on a retrospective cohort study which consists of COVID-19 patients confirmed in the Gyeongnam Province from the start of the vaccination drive (February 2021) to 30 November 2021 (N = 10,879). In Korea, contact tracing on confirmed COVID-19 patients have been actively implemented and all people classified as close contacts of COVID-19 patients were immediately required to undergo PCR testing at designated screening clinics. Additionally, any citizen who wanted to get a PCR test because of symptom onset or personal opinion could get tested at the screening clinics until 2 February 2022. For any reason, detailed epidemiological investigations by administrative experts such as public health officers were conducted on all PCR-positive confirmed COVID-19 patients. Through a phone interview, a trained interviewer collected the demographic and clinical characteristics including age, sex, nationality, infection route, comorbidity, symptoms, and contact tracing information of patients as well as personal information. Although detailed information was collected through the investigation, this study was performed based on the summary data containing only limited information necessary to monitor the scale and epidemiological characteristics of COVID-19 cases in the Gyeongnam Province.

SARS-CoV-2 vaccination started in February 2021 for inpatients, residents and workers under the age of 65 years in nursing hospitals and nursing facilities. Then, the vaccination for the elderly aged ≥65 years began in May 2021 and was later expanded to other age groups in South Korea. In adolescents and children, vaccination was not started until October and the proportion of full vaccination was less than 25% compared to about 80% of full vaccination in adults until November which is the end of the study period. Therefore, we considered that there may be a difference in traits of vaccinated adults and vaccinated adolescents and children and excluded the vaccinated population younger than 20 years. Additionally, we excluded patients with incomplete information on SARS-CoV-2 vaccination, symptoms, and cycle of threshold (Ct) values for the RNA-dependent RNA polymerase (RdRp) and envelope (E) genes. A total of 9,030 confirmed COVID-19 patients were included in this study after applying exclusion criteria. The study protocol was approved by the Institutional Review Boards of the Gyeongsang National University Changwon Hospital (IRB approval no. 2021-12-031), and the study was performed in accordance with the principles of the Declaration of Helsinki. Since the data used in this study was collected as part of an epidemiologic investigation, individual informed consent was waived.

### Operative definition

COVID-19 patients with symptoms were defined as those who had at least one symptom, including fever, cough, sputum, sore throat, dyspnea, myalgia, ageusia, anosmia, chills, and rhinorrhea, at the time of real-time PCR (RT-PCR) confirmation of SARS-CoV-2 infection. Diabetes mellitus, hypertension, cardiovascular disease, chronic kidney disease, cancer, neurological disorder, cerebrovascular disease, liver disease, and chronic pulmonary disease were considered high-risk comorbidities.

We categorized the Ct values of *RdRp* and *E* genes to classify study subjects according to viral load level. Since there is no general criterion for cut-off of Ct values for low, moderate and high viral load, we checked the distribution of Ct value of *RdRp* and *E* genes, and Ct values were classified into three groups based on approximations of the interquartile range (≥25, 15–25, and <15).

Four COVID-19 vaccines, namely BNT162b2, AZD1222, mRNA-1273, and Ad26.COV2.S, are used in South Korea (S1 Table). COVID-19 vaccination status was defined as follows: unvaccinated (not received vaccination or having received the first dose within 14 days preceding the confirmation of SARS-CoV-2 infection), partially vaccinated (≥14 days after the first vaccination dose or having received a second dose within 14 days preceding the confirmation of SARS-CoV-2 infection), and fully vaccinated (confirmation of SARS-CoV-2 infection ≥14 days after the second vaccination dose). In case of vaccinees who were administered the Ad26.COV2.S vaccine, which has a single-dose immunization regimen, the unvaccinated were those who had not been vaccinated whereas the partially and fully vaccinated were those who tested positive within 14 days and after 14 days from the date of vaccination.

### Selection of potential confounders

We selected variables associated with both COVID-19 vaccination status and outcomes (presence of symptom and viral load) in the univariable analysis as potential confounders and included these variables in the statistical model for adjustment. The selected potential confounders include age, sex, infection route (international travel, individual contact, local cluster, and under investigation), comorbidity (yes vs. no), and nationality (Korean national vs. foreign citizen).

Meanwhile, it is known that the peak viral loads of SARS-CoV-2 are reached at the time of symptom onset and then gradually decline. At about 7 days after the onset of symptoms, the viral load decreases to approximately half of the peak viral load, and the difference in the viral load between vaccinated and non-vaccinated patients becomes obvious at that time [17]. As the time after symptom onset is highly related to viral load as described above and we found significant difference in the duration from symptom to diagnosis according to vaccination status, we additionally adjusted the duration from symptom onset to diagnosis along with the abovementioned covariates when we evaluated the association between vaccination status and viral load. We categorized COVID-19 patients into three groups: all asymptomatic COVID-19 patients and symptomatic patients whose duration from symptom onset to COVID-19 confirmation is less than equal to 7 days and more than 7 days.

### Statistical analysis

Categorical variables and continuous variables in demographic and clinical characteristics were compared by vaccination status using the chi-square test and the analysis of variance (ANOVA) test, respectively. We evaluated the impact of SARS-CoV-2 vaccination on COVID-19 symptoms by estimating the relative risk (RR) and corresponding 95% confidence interval (95% CI) based on Poisson regression.

The analysis of covariance (ANCOVA) was used to compare the least square mean of Ct value according to the vaccination status, after adjusting for potential confounders. Moreover, the association between vaccination and the Ct values of the *RdRp* and *E* genes was assessed using Poisson regression.

As the viral load could be affected by age and because the proportion of vaccinated individuals is high in the older population, due to the prioritization of older adults in the national COVID-19 vaccination in South Korea, the effect of vaccination on symptoms and viral load was investigated in an age-stratified analysis (age groups: <40, 40–59, 60–79, and ≥80 years). All statistical analyses were performed using the SAS ver. 9.4 (SAS Institute Inc., Cary, NC). A two-tailed alpha error of 5% was the standard cutoff for statistical significance.

## Results

We compared the demographic characteristics, presenting symptoms and prevalence of comorbidities, and mean Ct values among COVID-19 patients according to the vaccination status (Table 1). Fully vaccinated COVID-19 patients were older, having comorbidities associated with a high risk of severe COVID-19 more, and less likely to have symptoms at the time of confirmation of SARS-CoV-2 infection than the unvaccinated or partially vaccinated groups (all *p*<0.01). Additionally, higher Ct values for the *RdRp* gene were observed in the fully vaccinated group compared to those in the unvaccinated and partially vaccinated groups (*p*<0.01).

**Table 1:** Demographics and clinical characteristics of COVID-19 patients by vaccination status.

Characteristics	Unvaccinated	Partially Vaccinated	Fully Vaccinated	*p*-value
	N (%)	N (%)	N (%)	
Age, years^1^	43.9 (15.1)	47.3 (15.7)	57.1 (18.0)	<0.01
Sex				
Male	4,110 (54.9)	435 (60.5)	466 (56.4)	0.01
Female	3,375 (45.1)	284 (39.5)	360 (43.6)	
Foreigners				
No	6,186 (82.6)	555 (77.2)	705 (85.4)	<0.01
Yes	1,299 (17.4)	164 (22.8)	121 (14.6)	
Infection route				
International travel	182 (2.4)	1 (0.1)	22 (2.7)	<0.01
Individual contact	3,443 (46.0)	363 (50.5)	259 (31.4)	
Local cluster	2,407 (32.2)	228 (31.7)	447 (54.1)	
Unknown	1,453 (19.4)	127 (17.7)	98 (11.9)	
Comorbidities				
No	6,172 (82.5)	565 (78.6)	542 (65.6)	<0.01
Yes	1,313 (17.5)	154 (21.4)	284 (34.4)	
Diabetes mellitus	426 (5.7)	50 (7.0)	80 (9.7)	<0.01
Hypertension	793 (10.6)	98 (13.6)	169 (20.5)	<0.01
Cardiovascular disease	125 (1.7)	17 (2.4)	28 (3.4)	<0.01
Chronic kidney disease	23 (0.3)	6 (0.8)	14 (1.7)	<0.01
Cancers	73 (1.0)	5 (0.7)	19 (2.3)	<0.01
Neurological disorder	59 (0.8)	7 (1.0)	78 (9.4)	<0.01
Cerebrovascular disease	72 (1.0)	6 (0.8)	33 (4.0)	<0.01
Liver disease	71 (1.0)	6 (0.8)	7 (0.9)	0.92
Chronic pulmonary disease	86 (1.2)	8 (1.1)	12 (1.5)	0.73
Symptoms				
No	2,314 (30.9)	206 (28.6)	351 (42.5)	<0.01
Yes	5,171 (69.1)	513 (71.4)	475 (57.5)	
Duration from symptom onset to infection confirmation^1^^,^ ^2^	1.9 (2.3)	1.8 (2.6)	1.5 (2.8)	<0.01
Ct value for *RdRp* gene^1^	21.0 (6.5)	21.7 (6.5)	22.4 (7.0)	<0.01
Ct value for *E* gene^1^	20.8 (6.3)	21.3 (6.3)	22.0 (7.0)	<0.01

Abbreviations: N, number; Ct, cycle threshold; RdRp, RNA-dependent RNA polymerase; *E*, envelope.

^1^Summarized into mean (standard deviation).

^2^Assigned to “0” in case of asymptomatic infection.

We assessed the association between COVID-19 vaccination status and the presence of COVID-19 symptoms, and found a decreased risk of symptomatic COVID-19 in the fully vaccinated group compared to that in the unvaccinated group (RR (95% CI) = 0.93 (0.87–0.99); S2 Table). In contrast, there was an increased risk of symptomatic COVID-19 in the partially vaccinated group compared to the unvaccinated group (RR (95% CI) = 1.05 (1.00–1.11)). Additionally, we evaluated the vaccine effectiveness for each listed symptom of COVID-19 (S2 Table). The risks of cough, sputum, and myalgia were lower in the fully vaccinated group than in the unvaccinated group. In contrast, higher risks of fever and rhinorrhea were observed in the fully vaccinated group (Table 2).

**Table 2:** Association between COVID-19 vaccination status and the presence of each symptom at diagnosis in the Gyeongnam Province.

Symptom	Vaccination	Symptom-absent	Symptom-present	RR (95% CI)^1^
		N (%)	N (%)	
**Fever**	Unvaccinated	5,433 (72.6)	2,052 (27.4)	1.00
	Partially vaccinated	552 (76.8)	167 (23.2)	0.90 (0.79–1.04)
	Fully vaccinated	622 (75.3)	204 (24.7)	1.15 (1.02–1.32)
**Cough**	Unvaccinated	5,508 (73.6)	1,977 (26.4)	1.00
	Partially vaccinated	514 (71.5)	205 (28.5)	1.09 (0.96–1.23)
	Fully vaccinated	655 (79.3)	171 (20.7)	0.86 (0.75–0.99)
**Sputum**	Unvaccinated	6815 (91.1)	670 (8.9)	1.00
	Partially vaccinated	671 (93.3)	48 (6.7)	0.78 (0.59–1.04)
	Fully vaccinated	774 (93.7)	52 (6.3)	0.74 (0.56–0.98)
**Sore throat**	Unvaccinated	5,615 (75.0)	1,870 (25.0)	1.00
	Partially vaccinated	543 (75.5)	176 (24.5)	1.04 (0.91–1.18)
	Fully vaccinated	681 (82.5)	145 (17.5)	0.89 (0.76–1.03)
**Dyspnea**	Unvaccinated	7,429 (99.2)	56 (0.8)	1.00
	Partially vaccinated	712 (99.0)	7 (1.0)	1.11 (0.50–2.46)
	Fully vaccinated	826 (100.0)	0 (0.0)	N/A
**Myalgia**	Unvaccinated	5,948 (79.5)	1,537 (20.5)	1.00
	Partially vaccinated	588 (81.8)	131 (18.2)	0.91 (0.78–1.07)
	Fully vaccinated	727 (88.0)	99 (12.0)	0.65 (0.53–0.79)
**Headache**	Unvaccinated	6,290 (84.0)	1,195 (16.0)	1.00
	Partially vaccinated	607 (84.4)	112 (15.6)	1.00 (0.84–1.20)
	Fully vaccinated	730 (88.4)	96 (11.6)	0.97 (0.80–1.18)
**Chills**	Unvaccinated	6,918 (92.4)	567 (7.6)	1.00
	Partially vaccinated	681 (94.7)	38 (5.3)	0.73 (0.53–1.00)
	Fully vaccinated	783 (94.8)	43 (5.2)	0.73 (0.54–1.00)
**Ageusia**	Unvaccinated	7,347 (98.2)	138 (1.8)	1.00
	Partially vaccinated	706 (98.2)	13 (1.8)	1.14 (0.65–2.01)
	Fully vaccinated	822 (99.5)	4 (0.5)	0.43 (0.16–1.77)
**Anosmia**	Unvaccinated	7,278 (97.2)	207 (2.8)	1.00
	Partially vaccinated	700 (97.4)	19 (2.6)	1.16 (0.73–1.84)
	Fully vaccinated	813 (98.4)	13 (1.6)	1.00 (0.57–1.77)
**Rhinorrhea**	Unvaccinated	7,113 (95.0)	372 (5.0)	1.00
	Partially vaccinated	634 (88.2)	85 (11.8)	2.52 (2.02–3.16)
	Fully vaccinated	758 (91.8)	68 (8.2)	1.83 (1.40–2.37)

Abbreviations: N, number; RR, relative risk; CI, confidence interval.

^1^Adjusted for age, sex, infection route, comorbidity (yes vs. no), and nationality (Koreans vs. foreigners).

We additionally investigated the impact of COVID-19 vaccination on symptoms in an age-stratified analysis (S3 Table). Though decrease of symptom onset in the fully vaccinated groups younger than 80 years (<40, 40–59, and 60–79 years), the statistical significance was not attained (RR (95% CI) = 0.92 (0.83–1.03) for <40 years; RR (95% CI) = 0.92 (0.82–1.03) for 40–59 years; RR (95% CI) = 0.93 (0.84–1.03) for 60–79 years, respectively).

**Fig 1:**
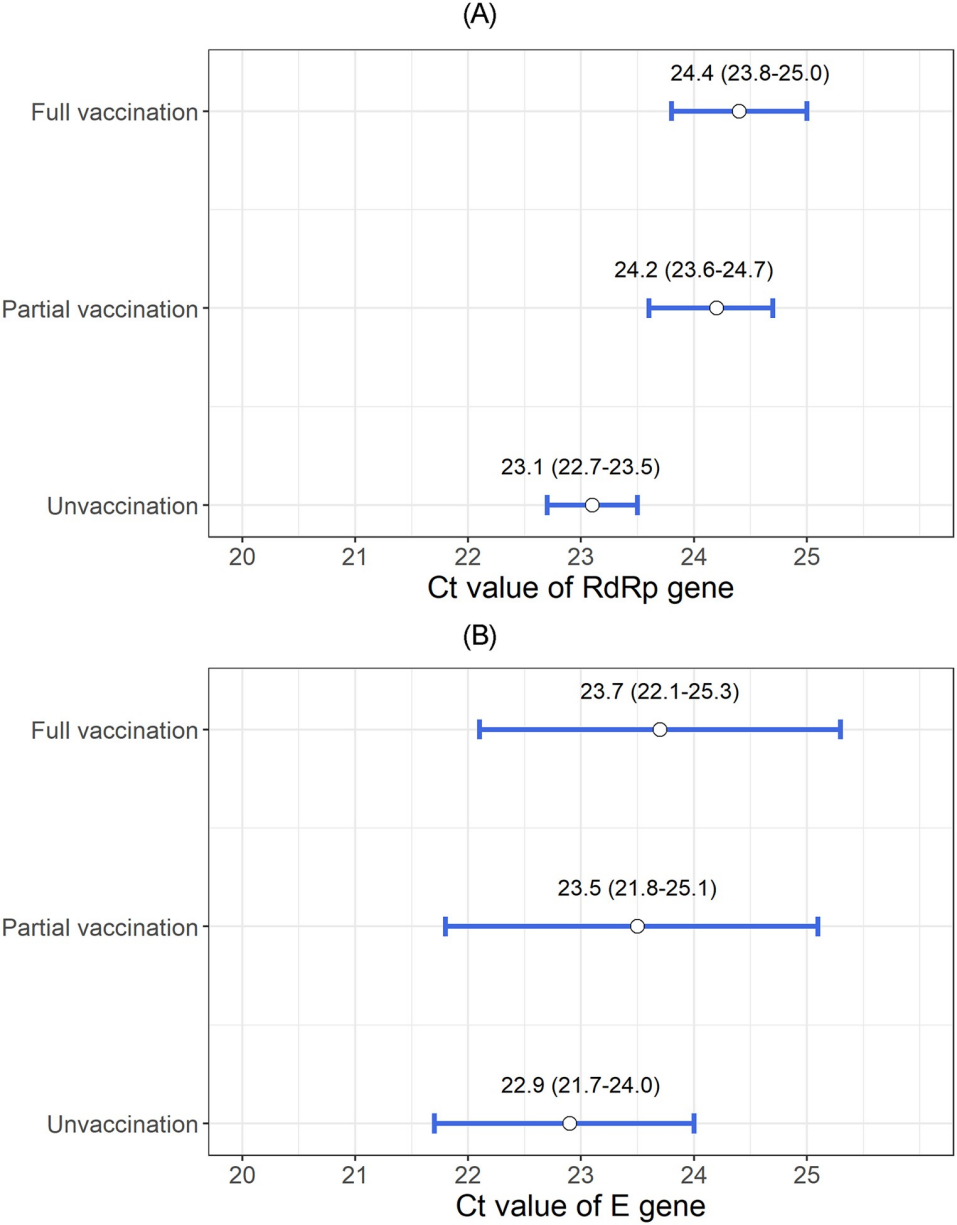
(A) Least squares mean and 95% confidence interval of the Ct value of the *RdRp* gene according to the COVID-19 vaccination status (B) Least squares mean and 95% confidence interval of the Ct value of the *E* gene according to the COVID-19 vaccination status.

Furthermore, we compared the least square means of the Ct values of the *RdRp* and *E* genes according to the COVID-19 vaccination status after adjusting for potential confounders. The highest Ct value of the *RdRp* gene was detected in the fully vaccinated group, and a sequential decrease in Ct values was found in the partially vaccinated and unvaccinated groups (mean, 95% CI: unvaccinated, 23.1, 22.7–23.5; partially vaccinated, 24.2, 23.6–24.7; and fully vaccinated, 24.4, 23.8–25.0) (Fig 1A). In post-hoc analysis, we found that the mean Ct value of *RdRp* gene in unvaccinated group was significantly lower than that in partially vaccinated group and fully vaccinate group. Sequential increase in the Ct value of the *E* gene was also observed in order of unvaccinated, partially vaccinated, and fully vaccinated patients (mean, 95% CI: unvaccinated, 22.9, 21.7–24.0; partially vaccinated, 23.5, 21.8–25.1; and fully vaccinated, 23.7, 22.1–25.3), however those differences were not statistically significant (Fig 1B).

The impact of COVID-19 vaccination on Ct values of the *RdRp* and *E* genes is presented in Table 3. Patients with low Ct value of the *RdRp* gene (15≤Ct<25) was lower in both partially vaccinated group (RR (95% CI) = 0.96 (0.91–1.02)) and fully vaccinated group (RR (95% CI) = 0.89 (0.83–0.95)) compared to unvaccinated group. When we additionally evaluated the association between vaccination status and the lowest level of Ct value of the *RdRp* gene (<15), the risk was reduced in both the partially vaccinated group (RR (95% CI) = 0.72 (0.60–0.86)) and fully vaccinated group (RR (95% CI) = 0.71 (0.61–0.84)). Similarly, the COVID-19 partial and full vaccination was associated with decreased viral loads of *E* gene, though the association was marginally significant for the full vaccination.

**Table 3:** Association between COVID-19 vaccination and the risk of high viral load in the Gyeongnam Province.

Vaccination status	25≤Ct (Controls)^1^ N (%)	15≤Ct<25 N (%)	RR (95% CI)^2^	15>Ct N (%)	RR (95% CI)^2^
**RdRp gene**					
Unvaccinated	2,047 (27.4)	4,132 (55.3)	1.00	1,287 (17.2)	1.00
Partially vaccinated	220 (30.8)	406 (56.9)	0.96 (0.91–1.02)	88 (12.3)	0.72 (0.60–0.86)
Fully vaccinated	295 (36.0)	408 (49.8)	0.89 (0.83–0.95)	116 (14.2)	0.71 (0.61–0.84)
**E gene**					
Unvaccinated	1,949 (26.1)	4,173 (56.0)	1.00	1,334 (17.9)	1.00
Partially vaccinated	214 (30.1)	394 (55.3)	0.95 (0.89–1.01)	104 (14.6)	0.79 (0.67–0.92)
Fully vaccinated	285 (34.7)	385 (46.9)	0.86 (0.81–0.93)	151 (18.4)	0.88 (0.76–1.01)

Abbreviations: Ct, cycle threshold; N, number; RR, relative risk; CI, confidence interval; RdRp, RNA-dependent RNA polymerase; E, envelope.

^1^Defined as COVID-19 patients with Ct value ≥25

^2^Adjusted for age, sex, infection route, comorbidity (yes vs. no), and nationality (Koreans vs. foreigners).

Furthermore, the COVID-19 vaccine effectiveness for preventing high viral loads was evaluated in an age-stratified analysis. The risk of the highest viral load of the *RdRp* gene (Ct <15) was lower in the fully vaccinated group in two age groups (20–39 years and 60–79 years) (S4 Table). The likelihood of the lowest Ct value of the *E* gene (Ct <15) significantly decreased in the partially vaccinated and fully vaccinated groups aged of 20–39 years (S5 Table).

## Discussion

In this study, we found that full vaccination helped prevent COVID-19 symptoms including cough, sputum, and myalgia in fully vaccinated COVID-19 patients. In addition, full vaccination was associated with a lower viral load in patient aged younger than 40 years.

In previous studies, the proportion of patients with symptoms or severe symptoms (pulse oxygen saturation <90% on room air, or respiratory rate >30 bpm, or features of severe disease such as shock or organ dysfunction) on arrival at the hospital was significantly lower in COVID-19 patients who had received COVID-19 vaccination than in unvaccinated COVID-19 patients [18, 19]. Furthermore, another study reported the symptom-prevention effectiveness of the COVID-19 vaccines, which ranged from a minimum of 49.5% to a maximum of 98.5% according to age groups and days from vaccination to confirmation of SARS-CoV-2 infection [20]. Similarly, we also found that vaccination prevented the occurrence of COVID-19 symptoms. This result may be a less biased estimate of the association between vaccination and presence of symptoms from those reported elsewhere [18–20], because we adjusted for potential confounders and performed age-stratified analyses. However, though we made an effort to control the effects of confounders, we found that fever and rhinorrhea were more prevalent in the vaccinated group compared to unvaccinated group. Therefore, further research is needed to elucidate whether these results are due to insufficient control of confounding factors or whether vaccination actually has a significant association with increased symptoms of fever and rhinorrhea. Although we cannot fully explain why fever and rhinorrhea symptoms are rather prevalent in fully vaccinated patients, it may be derived from the characteristics of fully vaccinated patients rather than the vaccine itself causing symptoms. According to a study reporting differences in symptom patterns according to age in COVID-19 patients, anorexia together with fever was the most common symptom pattern in the patients aged ≥65 years, while the top most symptom pattern in the patients aged of 45–64 years and aged <45 years were nausea together with weakness and dry mouth with sore throat, respectively [21]. In our study, the proportion of elderly was higher in the fully vaccinated group than other groups. Therefore, there would have been many patients with fever in the fully vaccinated group and this may lead to the increased risk of fever in the fully vaccinated group. However, rhinorrhea was discovered as a common symptom even in the patients aged <45 years in the same study, thus further research is needed to explain why the risk of rhinorrhea increased in the fully vaccinated patients.

Though we found little elevated risk of symptom onset in the partially vaccinated group, it is difficult to consider this as clinically meaningful increase. In addition, the symptom group included patients with various spectrums of symptoms, and rhinorrhea was the only symptom with a significantly increased risk in the partially vaccinated group. Therefore, interpretation of the increased risk of symptoms in the partial vaccination group requires caution.

Furthermore, previous studies have reported higher Ct values of *RdRp*, *E*, and *N* genes in vaccinated COVID-19 patients compared to those in unvaccinated patients, which is similar to the results of the present study [22, 23]. However, in these previous studies, potential confounders were not considered when comparing Ct values according to the vaccination status. In contrast, we compared the least square mean of Ct values after adjusting for age, sex, infection route, comorbidity, and nationality. Therefore, a less biased comparison of Ct values according to vaccination status was available in our study. In addition, we found that the vaccine was effective in preventing the highest viral load (Ct <15) in age groups younger than 40 years. However, in contrast to our findings, another study was reported that vaccination had no association with lower viral load [24]. Therefore, additional research is needed to establish the relationship between COVID-19 vaccination and viral load.

Despite the meaningful outcomes of this study, this study has several limitations. First, information on symptoms was telephonically collected only once from the primary epidemiological investigation based on self-report and only symptoms at the time of COVID-19 diagnosis were considered in this study. Therefore, the possibility of misclassification and underestimation of the symptoms might exist. However, this misclassification may have occurred randomly between the comparison groups (unvaccinated, partially vaccinated and fully vaccinated). Random misclassification usually leads the study results toward null, thus reducing the strength of the association between independent variable and dependent variable. Nevertheless, we were still able to find the significant association between the vaccination and the symptom occurrence. Second, the severity of symptoms, such as body temperature or O2 saturation, could not be investigated. Third, though Ct values were measured using RT-PCR kits from different manufacturers, we simply unified all Ct values, as if these values were derived from the same kit. A number of SARS-CoV-2 diagnostic kits based on the RT-PCR method were approved for use by the Ministry of Food and Drug Safety in Korea and there are studies for validation of the performance of these kits [25]. All these kits had the adequate performance with high sensitivity, however differences in Ct values were observed among kits for the same sample. This means that there is a possibility of misclassifying viral loads when simply integrating all Ct values from different kits. However, it is unlikely that a certain comparison group has a high percentage of using a particular RT-PCR kit, but rather it is likely that the use of the kit was randomly assigned among comparison groups. Non-differential misclassification leads to bias toward null, therefore difference in Ct values among kits would lead to the estimation between vaccination status and Ct value toward null value. Fourth, baseline characteristics among comparison groups (unvaccinated, partially vaccinated and fully vaccinated) were quite different. In Korea, vaccination began primarily for inpatients, residents, and workers in nursing hospitals and nursing facilities. Therefore, there were many COVID-19 patients who were older and with underlying diseases in the fully vaccinated group. In addition, there were many patients with a local cluster infection route and the duration from symptom onset to COVID-19 confirmation was short since many confirmed cases in the nursing hospital and nursing facility were discovered through test performed on all residents and workers when COVID-19 cases were found in that facility. Although we considered those differences in characteristics according to vaccination status in the model, it cannot be guaranteed that these differences were sufficiently adjusted.

Despite these limitations, this is the study to evaluate the effectiveness of vaccination on the occurrence of COVID-19 symptoms and viral load after the implementation of COVID-19 vaccination in real-world after controlling for many confounding factors. As this study used large province-based data from more than 9,000 COVID-19 patients, the results of this study may be applicable to the general population.

In conclusion, full vaccination against SARS-CoV-2 could be effective in the era of COVID-19 breakthrough infection increases through a reduction in the symptom occurrence and the viral load. This impact of vaccine on symptoms and viral load in COVID-19 patients could be used in the advocacy to minimize the tendency to vaccine hesitancy or refusal.

## Supporting information

S1 Table SARS-CoV-2 vaccine available in South Korea. (DOCX)

S2 Table Summary of association between COVID-19 vaccination status and symptoms at diagnosis in the Gyeongnam Province. (DOCX)

S3 Table Age-stratified association between COVID-19 vaccination status and symptoms. (DOCX)

S4 Table Age-stratified association between COVID-19 vaccination and Ct value of the RdRp gene. (DOCX)

S5 Table Age-stratified association between COVID-19 vaccination and Ct value of the E gene. (DOCX)
