# Supplementary material for: Vaccine effectiveness in symptom and viral load mitigation in COVID-19 breakthrough infections in South Korea
Source: PLoS One. 2023 Aug 16;18(8):e0290154. doi: 10.1371/journal.pone.0290154 (PMC10431655; doi:10.1371/journal.pone.0290154)
Supplement: S1 Table — (DOCX) [file pone.0290154.s001.docx]

**Supplementary Table 1**. SARS-CoV-2 vaccine available in South Korea

| **Vaccine name** | **Manufacturer** | **Vaccine classification** | **Immunization regimen** |
| --- | --- | --- | --- |
| BNT162b2 | Pfizer-BioNTech | mRNA | 30 μg (2 doses administered 3 weeks apart) |
| AZD1222 | AstraZeneca | Viral vector | 0.5 × 10^11^ viral particles  (2 doses administered 4 weeks apart) |
| mRNA-1273 | Moderna | mRNA | 100 μg (2 doses administered 4 weeks apart) |
| Ad26.COV2.S | Janssen | Viral vector | 5 × 10^10^ viral particles (1 dose) |
