# Supplementary material for: Vaccine effectiveness in symptom and viral load mitigation in COVID-19 breakthrough infections in South Korea
Source: PLoS One. 2023 Aug 16;18(8):e0290154. doi: 10.1371/journal.pone.0290154 (PMC10431655; doi:10.1371/journal.pone.0290154)
Supplement: S2 Table — (DOCX) [file pone.0290154.s002.docx]

**Supplementary Table 2**. Summary of association between COVID-19 vaccination status and symptoms at diagnosis in the Gyeongnam Province

| **Vaccination status** | **Symptom-absent** | **Symptom-present** | **RR (95% CI)^1^** |
| --- | --- | --- | --- |
|  | **N (%)** | **N (%)** |  |
| Unvaccinated | 2,314 (30.9) | 5,171 (69.1) | 1.00 |
| Partially vaccinated | 206 (28.7) | 513 (71.4) | 1.05 (1.00–1.11) |
| Fully vaccinated | 351 (42.5) | 475 (57.5) | 0.93 (0.87–0.99) |

Abbreviations: N, number; RR, relative risk; CI, confidence interval.

^1^Adjusted for age, sex, infection route, comorbidity (yes vs. no), and nationality (Koreans vs. foreigners).
