# Supplementary material for: Vaccine effectiveness in symptom and viral load mitigation in COVID-19 breakthrough infections in South Korea
Source: PLoS One. 2023 Aug 16;18(8):e0290154. doi: 10.1371/journal.pone.0290154 (PMC10431655; doi:10.1371/journal.pone.0290154)
Supplement: S3 Table — (DOCX) [file pone.0290154.s003.docx]

**Supplementary Table 3**. Age-stratified association between COVID-19 vaccination status and symptoms

| **Age, in years** | **Vaccination status** | **Symptom-absent** | **Symptom-present** | **RR (95% CI)^1^** |
| --- | --- | --- | --- | --- |
|  |  | **N (%)** | **N (%)** |  |
| 20≤Age<40 | Unvaccinated | 880 (28.0) | 2,265 (72.0) | 1.00 |
|  | Partially vaccinated | 77 (30.3) | 177 (69.7) | 1.00 (0.92–1.09) |
|  | Fully vaccinated | 74 (39.1) | 115 (60.9) | 0.92 (0.83–1.03) |
| 40≤Age<60 | Unvaccinated | 976 (31.0) | 2,175 (69.0) | 1.00 |
|  | Partially vaccinated | 67 (26.6) | 185 (73.4) | 1.10 (1.02-1.19) |
|  | Fully vaccinated | 95 (43.0) | 126 (57.0) | 0.92 (0.82-1.03) |
| 60≤Age<80 | Unvaccinated | 399 (37.2) | 673 (62.8) | 1.00 |
|  | Partially vaccinated | 59 (28.6) | 147 (71.4) | 1.06 (0.96–1.17) |
|  | Fully vaccinated | 138 (44.1) | 175 (55.9) | 0.93 (0.84–1.03) |
| ≥80 | Unvaccinated | 59 (50.4) | 58 (49.6) | 1.00 |
|  | Partially vaccinated | 3 (42.9) | 4 (57.1) | 1.36 (0.68–2.70) |
|  | Fully vaccinated | 44 (42.7) | 59 (57.3) | 1.21 (0.95–1.53) |

Abbreviations: N, number; RR, relative risk; CI, confidence interval.

^1^Adjusted for age, sex, infection route, comorbidity (yes vs. no), and nationality (Koreans vs. foreigners).
