# Supplementary material for: Vaccine effectiveness in symptom and viral load mitigation in COVID-19 breakthrough infections in South Korea
Source: PLoS One. 2023 Aug 16;18(8):e0290154. doi: 10.1371/journal.pone.0290154 (PMC10431655; doi:10.1371/journal.pone.0290154)
Supplement: S4 Table — (DOCX) [file pone.0290154.s004.docx]

**Supplementary Table 4**. Age-stratified association between COVID-19 vaccination and Ct value of the RdRp gene

| **Age, in years** | **Vaccination status** | **25≤Ct**  **(Controls)^1^**  **N (%)** | **15≤Ct<25**  **N (%)** | **RR (95% CI)^2^** | **15>Ct**  **N (%)** | **RR (95% CI) ^2^** |
| --- | --- | --- | --- | --- | --- | --- |
| 20≤Age<40 | Unvaccinated | 843 (26.9) | 1,767 (56.3) | 1.00 | 528 (16.8) | 1.00 |
|  | Partially vaccinated | 73 (28.9) | 149 (58.9) | 0.98 (0.89–1.07) | 31 (12.2) | 0.73 (0.55–0.97) |
|  | Fully vaccinated | 64 (34.0) | 103 (54.8) | 0.97 (0.86–1.08) | 25 (11.2) | 0.69 (0.49–0.96) |
| 40≤Age<60 | Unvaccinated | 887 (28.2) | 1,722 (54.8) | 1.00 | 533 (17.0) | 1.00 |
|  | Partially vaccinated | 89 (35.9) | 140 (56.4) | 0.88 (0.80-0.98) | 19 (7.7) | 0.47 (0.31-0.70) |
|  | Fully vaccinated | 76 (34.7) | 112 (51.1) | 0.91 (0.81-1.03) | 31 (14.2) | 0.84 (0.61-1.16) |
| 60≤Age<80 | Unvaccinated | 282 (26.4) | 588 (55.0) | 1.00 | 199 (18.6) | 1.00 |
|  | Partially vaccinated | 57 (27.7) | 112 (54.3) | 0.97 (0.86–1.09) | 37 (18.0) | 0.94 (0.71–1.25) |
|  | Fully vaccinated | 111 (35.9) | 151 (48.9) | 0.86 (0.77–0.96) | 47 (15.2) | 0.72 (0.55–0.94) |
| ≥80 | Unvaccinated | 35 (29.9) | 55 (47.0) | 1.00 | 27 (23.1) | 1.00 |
|  | Partially vaccinated | 1 (14.3) | 5 (71.4) | 1.38 (0.89–2.13) | 1 (14.3) | 1.14 (0.29–4.45) |
|  | Fully vaccinated | 44 (42.7) | 42 (40.8) | 0.79 (0.60–1.04) | 17 (16.5) | 0.65 (0.39–1.11) |

Abbreviations: Ct, cycle threshold; N, number; RR, relative risk; CI, confidence interval; RdRp, RNA dependent RNA polymerase

^1^Defined as COVID-19 patients with Ct value ≥25

^2^Adjusted for age, sex, infection route, comorbidity (yes vs. no), and nationality (Koreans vs. foreigners).
