# Supplementary material for: Vaccine effectiveness in symptom and viral load mitigation in COVID-19 breakthrough infections in South Korea
Source: PLoS One. 2023 Aug 16;18(8):e0290154. doi: 10.1371/journal.pone.0290154 (PMC10431655; doi:10.1371/journal.pone.0290154)
Supplement: S5 Table — (DOCX) [file pone.0290154.s005.docx]

**Supplementary Table 5.** Age-stratified association between COVID-19 vaccination and Ct value of the E gene

| **Age, in years** | **Vaccination status** | **25≤Ct**  **(Controls)^1^**  **N (%)** | **15≤Ct<25**  **N (%)** | **RR (95% CI)^2^** | **15>Ct**  **N (%)** | **RR (95% CI) ^2^** |
| --- | --- | --- | --- | --- | --- | --- |
| 20≤Age<40 | Unvaccinated | 796 (25.5) | 1,750 (56.0) | 1.00 | 582 (18.6) | 1.00 |
|  | Partially vaccinated | 69 (27.6) | 142 (56.8) | 0.97 (0.889–1.07) | 39 (15.6) | 0.81 (0.64–1.03) |
|  | Fully vaccinated | 64 (34.0) | 99 (52.7) | 0.93 (0.83–1.05) | 25 (13.3) | 0.71 (0.53–0.95) |
| 40≤Age<60 | Unvaccinated | 837 (26.6) | 1,766 (56.3) | 1.00 | 536 (17.1) | 1.00 |
|  | Partially vaccinated | 91 (36.6) | 132 (53.0) | 0.83 (0.75-0.93) | 26 (10.4) | 0.56 (0.40-0.79) |
|  | Fully vaccinated | 77 (35.3) | 103 (47.3) | 0.86 (0.76-0.98) | 38 (17.4) | 0.95 (0.72-1.26) |
| 60≤Age<80 | Unvaccinated | 277 (25.8) | 605 (56.5) | 1.00 | 190 (17.7) | 1.00 |
|  | Partially vaccinated | 52 (25.2) | 116 (56.3) | 1.00 (0.89–1.11) | 38 (18.5) | 1.01 (0.77–1.33) |
|  | Fully vaccinated | 105 (33.7) | 140 (44.8) | 0.84 (0.75–0.94) | 67 (21.5) | 0.97 (0.78–1.21) |
| ≥80 | Unvaccinated | 39 (33.3) | 52 (44.5) | 1.00 | 26 (22.2) | 1.00 |
|  | Partially vaccinated | 2 (28.6) | 4 (57.1) | 1.22 (0.62-2.41) | 1 (14.3) | 0.86 (0.16-4.55) |
|  | Fully vaccinated | 39 (48.8) | 43 (41.8) | 0.92 (0.70–1.21) | 21 (20.4) | 0.94 (0.59–1.50) |

Abbreviations: Ct, cycle threshold; N, number; RR, relative risk; CI, confidence interval; E, envelope

^1^Defined as COVID-19 patients with Ct value ≥25

^2^Adjusted for age, sex, infection route, comorbidity (yes vs. no), and nationality (Koreans vs. foreigner)
